# Climate of doubt: A re-evaluation of Büntgen and Di Cosmo’s environmental hypothesis for the Mongol withdrawal from Hungary, 1242 CE

**DOI:** 10.1038/s41598-017-12128-6

**Published:** 2017-10-05

**Authors:** Zsolt Pinke, László Ferenczi, Beatrix F. Romhányi, József Laszlovszky, Stephen Pow

**Affiliations:** 1 Department of Nature Conservation and Landscape Ecology, Szent István University, Gödöllő, H-2100 Hungary; 2 Department of Medieval Studies, Central European University, Budapest, H-1051 Hungary; 3 Lendület’ Hungary in Medieval Europe Research Group LP2014-13/2014, MTA-University of Debrecen, Debrecen, H-4032 Hungary

## Abstract

In their recent article published in the journal *Scientific Reports*, Büntgen and Di Cosmo have attempted to solve the historical mystery of the sudden Mongol withdrawal from Hungary after a year-long occupation. We cannot share the authors’ viewpoint that environmental circumstances contributed to the decision of the Mongols to abandon Hungary since the hypothesis lacks support from environmental, archaeological and historical evidence. Historical source material in particular suggests that the Mongols were able to settle and sustain their herds in Hungary as is clearly stated in a letter by King Bela IV to the pope. The Mongol army arrived in the kingdom at the end of a severe drought, and we present empirical evidence that the abundant rain in the spring of 1242 CE did not worsen but rather improved their prospects for sufficient food supplies and pasturage. The marshy terrain of the Hungarian Plain likely did not precipitate the Mongol withdrawal as the Mongol high command ultimately stationed their main forces around the marshy Volga Delta. In contrast to what Büntgen and Di Cosmo have suggested, we argue that the reasons for the sudden withdrawal cannot be explained largely by environmental factors.

## Introduction

Büntgen and Di Cosmo’s recent article^1^ in *Scientific Reports* (2016) attempts to tackle an important historical mystery (the abrupt Mongol withdrawal from medieval Hungary) by employing techniques from the burgeoning field of climate science. We agree with their underlying assumption that an interdisciplinary analysis of environmental and documentary resources can result in a better understanding of the events. For example Pederson *et al*. and Putnam *et al*. demonstrate that the emergence of the Mongol Empire is likely tied to an increasingly wet climate in Inner Asia during the period^2,3^. However, as regards the conclusion of Büntgen and Di Cosmo’s study, some of the supporting evidence does not withstand critical examination in the context of the Mongol invasion of Hungary.

The key argument of the study is the “environmental hypothesis” which ascribes the Mongol withdrawal to the “general syndrome in which the effectiveness of nomadic armies was constrained by a short-term, regional-scale climate fluctuation”. Partly based on dendroclimatological evidence, the authors argue that the wet early-spring in 1242 CE, and the melting of snow which followed the unusually bitter winter, might have reduced the productivity of pastures as well as the manoeuvring capability of the nomadic invaders. They assert that “most of the lower elevation soil types in Hungary are particularly prone to stagnant moisture and ponding”. So, when the evident warm and dry summers of 1238–1241 were followed by cold and wet conditions in early 1242, “marshy terrain across the Hungarian plain most likely reduced pastureland and decreased mobility, as well as the military effectiveness of the Mongol cavalry”. This hypothesis is not unreasonable and merits consideration, but problems emerge in the way that both paleoclimatic data and material from historical sources are presented as evidence *supporting* the hypothesis. We will look at these aspects of the argument in turn.

## Environmental Arguments

**Figure 1:**
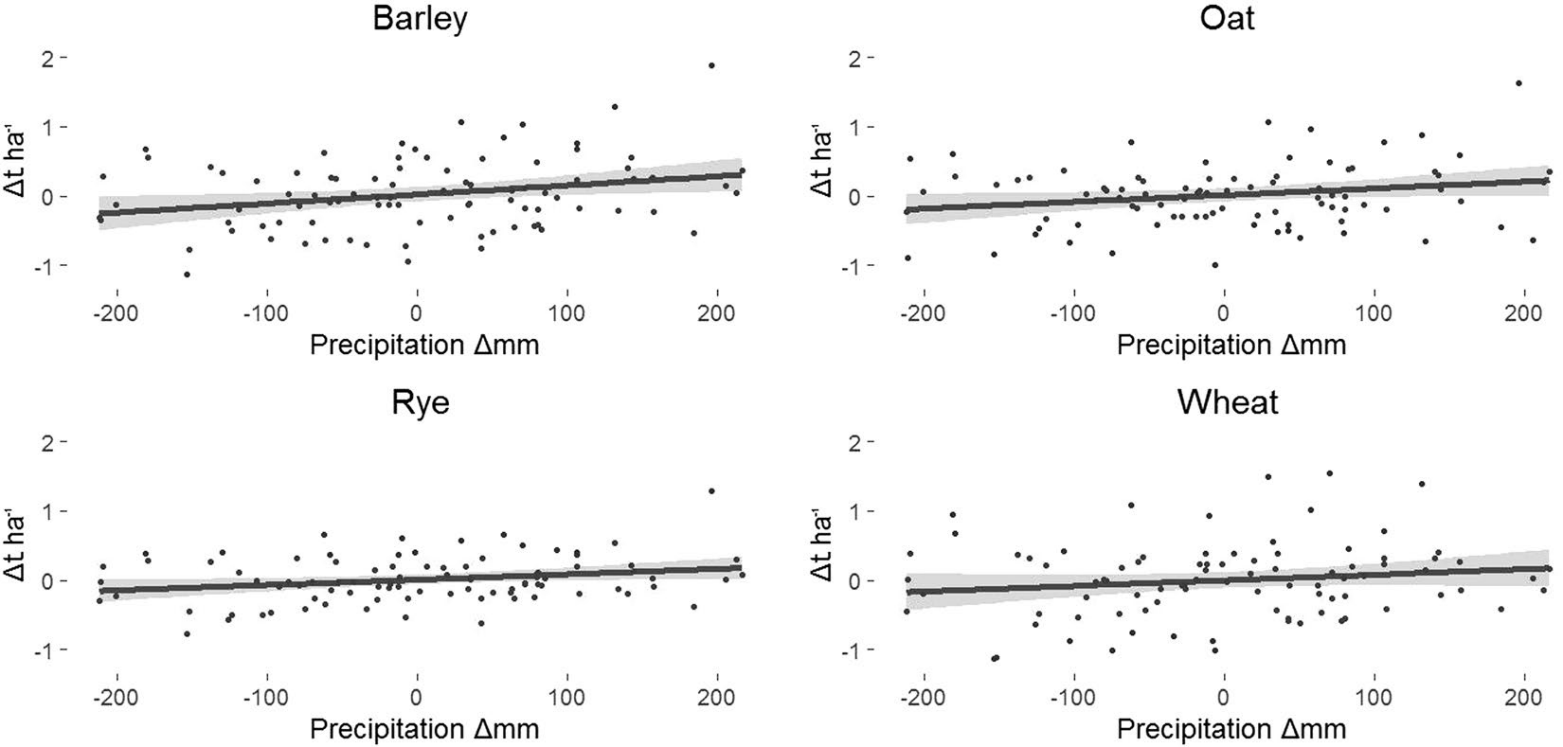
Multiple scatter plot for the linear relationship between the first-differences of March–August precipitation sums and the annual yield means of four crops in the period 1921–2010^29^. Precipitation shows significantly positive, temperature trend-like negative linear relationship with studied crop yields (See: Supplementary).

The authors, Büntgen and Di Cosmo, argue that a wet spell on the Hungarian plain in early 1242 reduced the nomadic Mongols’ military capability. Despite the authors’ viewpoint, much evidence points to the fact that springtime inundations on the drought-prone Hungarian plain have historically been beneficial for the grazing of its enormous livestock herds^4^. There is no evidence to support a statement which is crucial to their theory: “Soil wetness […] not only delays the onset of the vegetation period but also reduces the overall productivity of the extensive agricultural and natural grassland habitats in Hungary”. Recent research in landscape ecology shows quite the opposite. The first phase of inundations raises the grass yield of the grasslands in the temperate zone, while prolonged water coverage of pastures provides high fodder yields, reducing the ratio of forage grasses and increasing that of aquatic species^5^. As for the effect of the described climatic scenario on extensive agriculture, the relatively low precipitation period followed by a wet spring should have resulted in a significant growth in crop yields at a countrywide scale, even with instances of the disruption of agricultural activities at the local level due to serious flooding. Although a number of environmental variables may influence the productivity of crops significantly (e.g. floods, frost, pests), due to the climatic and geomorphological conditions of the Carpathian Basin before 19^th^ century agro-modernisation, it was droughts that caused serious crop losses at a countrywide scale. Historical studies on land use confirm that permanent saturation of soils due to floods or long-term rainfalls hindering crop development occurred in relatively small areas, since land users generally avoided flood prone areas for arable farming^6,7^. There are no data to demonstrate that wet climate conditions led to starvation in the entire Carpathian Basin. On the contrary, there is a qualitative analogy from the period 1921–2010 in which a positive correlation is evident between March–August precipitation and the yields of four important crops in Hungary (Fig. 1). While we would like to emphasize that there are no quantitative data on precipitation for 1241–1242 that allow a valid comparison of 13^th^ and 20^th^ century conditions, medieval and early modern data, as well as figures from the last 90 years, suggest that rising precipitation is positively correlated with crop yields in Hungarian agriculture.

Thus, the paper’s conclusion that, besides the effects of war, “a less favourable climate may have also caused the failure of the harvest of 1242 and the ensuing ‘great famine’ in Hungary” is not supported by the climate data. Medieval writers ascribed the famine to farmers being unable to plant crops or reap harvests for two growing seasons. As a contemporary observer noted: “For with the fury of the Tatars upon them, the poor farmers had not been able to plant the fields, nor could they bring in the previous harvest”^8^. Historical sources indicate that survivors of the occupation were displaced as refugees and often forced to hide in mountainous regions for long periods^9^. They also emphasize that the Mongols left Hungary with loaded wagons after looting the country. They had plundered the livestock and horses of Hungary to such an extent that Hungarian peasants were forced to attach ploughs to themselves as the country had few remaining draught animals. In short, the famine was man-made, deliberately or incidentally triggered by the activity of the Mongols, and was not at all a result of the climatic conditions. No source from the period suggests otherwise.

The assertion made by Büntgen and Di Cosmo^1^ that “reduced victuals for the army” caused the Mongol withdrawal, which implies that the famine which affected the local resident population was also forcing the nomadic Mongols into a food crisis, is questionable. This contradicts numerous sources written by observers across Eurasia which state that the Mongols relied primarily on their own herds for food^3,10^. So, even if Mongols did face a scarcity of local agricultural products, it does not prove that there was a shortage of forage or that the Mongols no longer had animals to rely on for food.

The paper “raises the possibility that the vulnerability of the Hungarian plains to even relatively short-term climate events made it obvious [to Mongol leaders] the region was unsuitable” for their pastoral nomadism, owing to the rivers’ susceptibility to flooding. The idea that the climate and marshy environment of Hungary were too inhospitable for Inner Asian nomads is difficult to accept. It is well documented that the Cumans, who were traditionally nomadic but increasingly moving to a sedentary lifestyle, tended to settle in areas which included the floodplains of the major rivers of the Hungarian plain after the Mongol invasion^11^. Thus, we should conclude that marshy terrains cannot have been a decisive factor in the Mongols’ decision to withraw^12^. But even if we were to entertain the possibility that Mongol military leaders laid great emphasis on environmental considerations, Büntgen and Di Cosmo’s argument cannot answer the following questions. Why then did the Mongols invade Hungary following four years of drought? Why did they leave during a wet period in 1242 when they could expect increased yields and fodder? Why did they not conquer Hungary and Poland, which had higher grass yields and carrying capacity than the marshy region of the southern Volga basin where Batu Khan established his capital? Why did they continue to wage an ongoing war of conquest in the forest zone of Russia after 1242, and launch a major invasion of the desert region of Iraq in the following decade? These questions receive speculative answers. However, what we would like to do is rely on specific concrete evidence for argumentation which we will provide in the following section.

The argument that the medieval Hungarian Kingdom provided insufficient resources for the Mongols’ enormous herds first appeared in Sinor’s paper^13^, and while Büntgen and Di Cosmo’s article does not follow an identical line of reasoning, we feel it is important here to refute any such viewpoint. The 110,000 km^2^ Hungarian Plain was the target area for waves of nomadic herders from the Eurasian steppe zone for thousands of years, including the Huns who, eight centuries before the Mongol invasion, relied on a nomadic cavalry force^14^. Much later, in the 16^th^ and 17^th^ centuries, the Ottomans occupied the middle section of the Carpathian Basin, including most of the Hungarian Plain. Hundreds of thousands of Ottoman occupiers, tens of thousands of European soldiers, and the local population were residing simultaneously in Hungary, so the numbers of troops involved may have outnumbered the Mongol invaders of the 13^th^ century^15^. It should be added that the Ottoman Empire occupied Hungary during a period of increasingly extreme weather conditions, precipitated by one of the most severe periods of what historians and climatologists now dub the Little Ice Age. Despite the clear effects on local people, the occupying armies’ ability to sustain themselves and operate was not seriously affected^16^. Admittedly, these later armies did not place the same emphasis on cavalry, but fortunately, it is precisely from the 16^th^ century that we first obtain figures on the numbers of livestock grazing in Hungary. The Hungarian and Polish Kingdoms became the two largest cattle exporters of Europe so that, by the sixteenth century, Hungary – primarily its lowland areas – regularly supplied herds of cattle that occasionally totalled over 200,000. Exporting cattle on this scale could only be sustained if millions of animals were continually grazing. The individual body mass of these animals was much bigger than that of a nomadic horse^17,18^. Thus, their forage demand was much higher than that of the nomadic horses of the Mongols. In the eighteenth century, detailed information from livestock censuses indicate that in just one 200 km^2^ pasture near the city of Debrecen (ca. 0.2% of the Hungarian Plain), 13,500 cattle, 1,100 horses, and 18,000 sheep were registered in 1770–72^19^. In 1861, an area comprising six Cuman settlements (ca. 1% of the Hungarian plain) documented the presence of 30,593 heads of cattle, 15,190 horses, 245,340 sheep, and 26,100 pigs^20^. Historical ethnographic research confirmed that animal husbandry in the two cited years was characterized by the same practices used in the Middle Ages^21^. One may rightly presume that the 330,000 km^2^ Hungarian Kingdom – of which ca. 40% was plain – could have sustained millions of horses in the thirteenth century. Indeed, medieval sources seem to indicate the Mongol army had sufficient forage for its horses. After the withdrawal, King Béla IV (1235–1270 CE) clearly stated in a letter to the pope that Hungary was suited for the Mongols and their enormous herds: “They can settle their families and animals – in which they abound – marvellously well here [in Hungary], better than elsewhere”^22^. Thus, there is little evidence to support the argument that insufficient fodder played a role in the abrupt Mongol withdrawal. In general, theories on the Mongol withdrawal based on ecological considerations tend to grossly downplay the carrying capacity of the Carpathian Basin, especially when we consider that the Mongols’ steppe horses were well-known for their endurance and ability to survive on little food^8^.

## Documentary Evidence

Since Büntgen and Di Cosmo’s environmental hypothesis holds that climatic changes in early 1242 did not allow Mongols “to function effectively as an occupation army, thus forcing them to withdraw”, it establishes the premise that the Mongols faced difficulties mainly in 1242 when they experienced, for instance, setbacks taking fortresses after crossing to the western side of the Danube River. This viewpoint does not take into account that Mongol-Chinese government sources record that Batu wanted to withdraw from Hungary during the Battle of Mohi and was dissuaded from retreat by his fellow commander, Subutai^23^. Such a premise also requires us to overlook the crucial question of why it took the Mongols nine months to first cross the Danube after defeating the Hungarians at Mohi, only some 160 km east of the river. Sources mention that internal divisions in the kingdom caused many Hungarian nobles to ignore the call to assemble their forces before the battle, so a significant part of the Hungarian army escaped destruction and thus was able to defend western Hungary. Despite the fact that 1241 was a drought year, and the fords of the Danube could be waded on horseback during dry summers and autumns, the Mongols were prevented from crossing. Yet, their skill at crossing rivers was widely noted and they previously successfully crossed the largest rivers of northern Eurasia which are much wider and more dangerous than the Danube. The sources offer little detail on this aspect of the invasion, but enough to conclude that Hungarian resistance played a role in stopping the Mongols from crossing before early 1242 when the river froze. One author who was taken prisoner by the Mongols, Rogerius, states that the Hungarians regularly broke the ice and guarded the river so that “the foot soldiers continuously fought on the ice”^9^. King Béla IV also wrote a letter to the pope, explaining his decision to fortify the Danube line on the basis that his troops had halted the invaders along the “water of resistance” for ten months after the disaster at Mohi^22^. Therefore, armed resistance may have played a role in hindering the Mongol advance.

On this point, we would like to add archaeological evidence which appears to refute the hypothesis of Büntgen and di Cosmo. In recent times, there has been an unexpected increase in the number of sites which reveal direct evidence of the Mongol invasion, and their locations strongly suggest that the most heavily affected areas, in terms of destruction, were in the Great Plain in the eastern and central part of the country^24^, along with the stretch of plains immediately west of the Danube. Therefore, we see no evidence that Mongol forces were impeded in the plain areas which were most affected by wet conditions. Finds of coin hordes, dating from 1241–1242, are found primarily in the same regions as well, supporting this viewpoint^25^.

**Figure 2:**
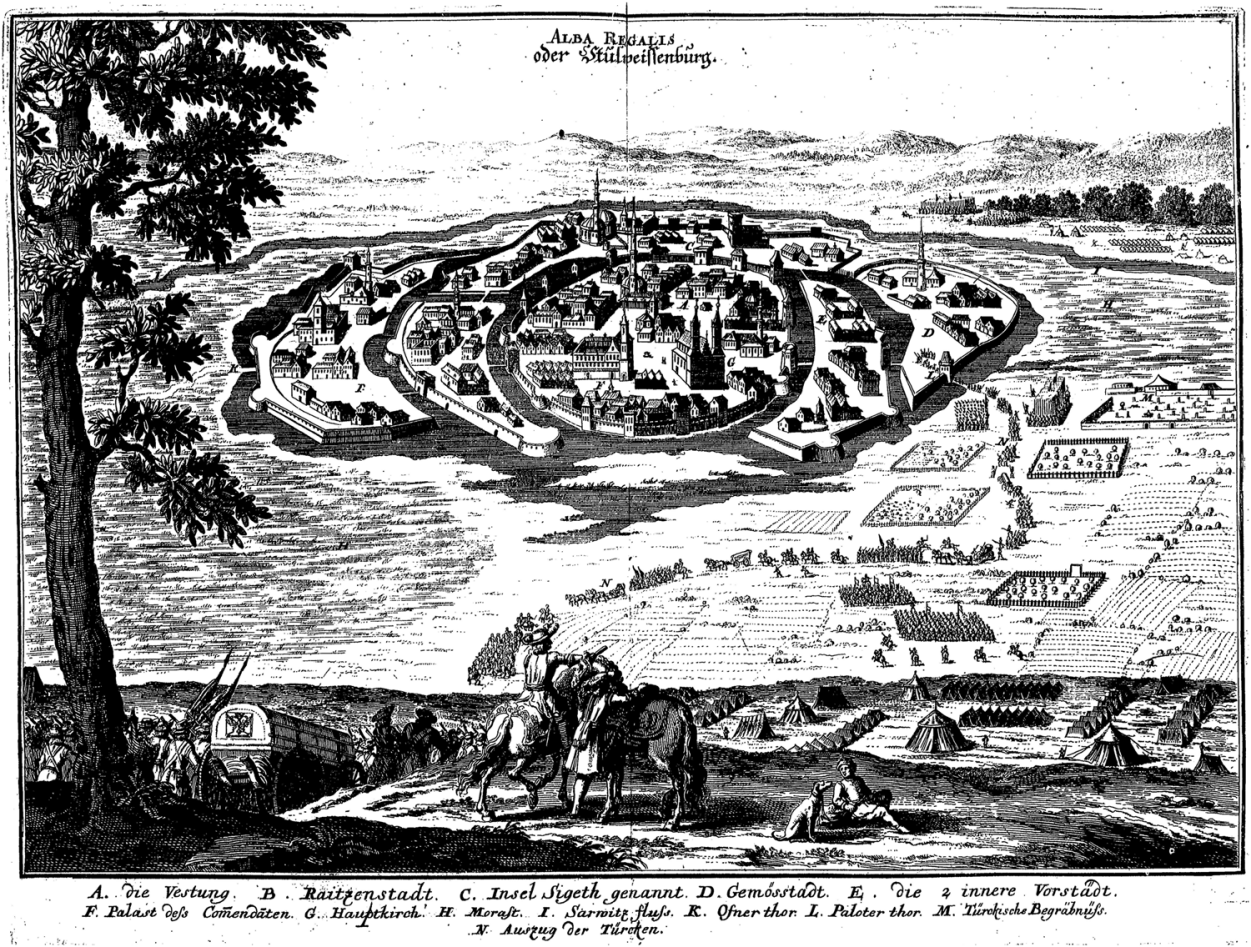
Székesfehérvár in the seventeenth century. An Early Modern depiction of the city before the modern drainage activity, shows that it was ordinarily surrounded by marshes. The image is from a 1667 print of Merian’s *Theatrum Europaeum* ^27^.

Büntgen and Di Cosmo pay special attention to Mongol attempts to take Székesfehérvár and Trogir in spring 1242. These two examples are intended to support a contention of the environmental hypothesis that wet, muddy conditions foiled assaults on fortified sites. The written references to these attacks do not strongly support this hypothesis. Székesfehérvár, like other fortresses situated on the Hungarian lowlands, was built on islands surrounded by wetlands and marshes to inhibit the advance of any hostile force. Early modern drawings reveal that the town was surrounded by marshes (Fig. 2), and medieval settlement history reveals that this was the permanent situation^26^. Thus, the account of marshy conditions exacerbated by melting ice in spring 1242 did not represent an unusual situation. Several Mongol attacks on island-like wetland fortresses like Székesfehérvár, or Breslau in Poland, ended abortively. Regarding Trogir in Croatia, the suggestion that heavy rain caused the muddy conditions which foiled Mongol attacks could be misleading because Trogir is a coastal island separated from the mainland by a channel of sea water rather than a freshwater moat. In the case of two other unsuccessful Mongol sieges, at Esztergom and Pannonhalma, the sources do not attribute the failure to wet weather and spring runoff, but to the active resistance of the besieged.

## Discussion

Büntgen and Di Cosmo^1^ argue that the climatic trends of 1241–1242 worked against the Mongols, playing a large part in bringing about their withdrawal. Interestingly, one could justifiably argue the opposite – namely, that climatic conditions in 1241–1242 greatly facilitated the Mongol occupation of Hungary because wet and cold conditions appear to have aided the Mongols and worked against the Hungarians at two crucial points during the campaign. First, at the Battle of Mohi, “almost the whole” of the fleeing Hungarian army drowned in the enormous nearby swamp while trying to escape their pursuers. Second, the unusual cold during the winter of 1241–1242 froze the Danube which finally enabled the Mongol forces to cross the ice and attack the hitherto unoccupied parts of the country. Such an argument, however, would not address the historical mystery of the withdrawal.

Research on environmental changes and their effects on past societies necessitates the continued development of interdisciplinary approaches, and as Putnam *et al*.^3^ suggested, the investigation of the impact of changing climate and landscape conditions on the rise of the Mongol Empire is a worthwhile endeavour. Highlighting the drivers behind the rise and fall of Eurasian steppe societies, which always depended on animal husbandry, will benefit from the contributions of many disciplines, such as anthropology, archaeology, climatology, history, landscape ecology, etc. Moreover, short-term climatic fluctuations can indeed alter human activity and the course of history^28^. Büntgen and Di Cosmo^1^ offer important regional-scale reconstructions of temperatures in thirteenth century Central Europe. However, these do not explain the Mongol withdrawal, and the mystery remains.

### Data availability statement

The datasets analysed during the current study (see Fig. 1 and Supplementary Fig 1) are available in the Hungarian Central Statistical Office and Hungarian Meteorological Service’s repository, (https://www.ksh.hu/docs/hun/agrar/html/tabl1_4_3_1.html;.

## Electronic supplementary material

Supplementary

## References

[1] Büntgen U, Di Cosmo N (2016). Climatic and environmental aspects of the Mongol withdrawal from Hungary in 1242 CE. Scientific Reports.

[2] Pederson N, Hessl AE, Baatarbileg N, Anchukaitis KJ, Di Cosmo N (2014). Pluvials, droughts, the Mongol Empire, and modern Mongolia. Proc. Natl. Acad. Sci. USA..

[3] Putnam AE (2016). Little Ice Age wetting of interior Asian deserts and the rise of the Mongol Empire. Quat. Sci. Rev..

[4] The regulation of the Council of Túrkeve in 1780 published by Györffy, I. Nagykunsági krónika [Chronical of Nagykunság]. (Szépirodalmi könyvkiadó, 1955).

[5] Tasi J (2012). Relationship of feed quality and water supply on dry and mesic pastures. Növényterm..

[6] Bellon, T. B. A nagykunsági mezővárosok állattartó gazdálkodása a XVIII-XIX. században [Beklen. The economy of Animal Husbandry Market Towns in Nagykunság Region]. (Karcag Város Önkormányzata, 1996).

[7] Pinke (2017). Zonal assessment of environmental driven settlement abandonment in the medieval Trans-Tisza region, Central Europe. Quat. Sci. Rev..

[8] Thomas of Split. History of the Bishops of Salona and Split (eds Karbić, D. *et al*.) 302–303 (Central European University Press, 2006).

[9] Anonymous and Master Roger. Magistri Rogerii Epistula miserabile carmen super destruction regni Hungariae per Tartaros facta (eds. Bak, J. M. & Rady, M.) 201; 205; 222–225 (Central European University Press, 2010).

[10] Ibn al-Athir. The Chronicle of Ibn al-Athir for the Crusading Period from al-Kamil fi’l-ta’rikh. Part 3: The Years 589-629/1193–1231: The Ayyubids after Saladin and the Mongol Menace (trans. Richards, D.S.) 203 (Ashgate, 2003).

[11] Lyublyanovics, K. The Socio-Economic Integration of Cumans in Medieval Hungary. An Archaeozoological Approach Doctoral Dissertation. (Central European University, 2015).

[12] Fletcher J (1986). The Mongols: Ecological and Social Perspectives. Harv. J. Asiat. Stud..

[13] Sinor D (1972). Horse and Pasture in Inner Asian History. Oriens Extremus.

[14] Priscus of Panium. The Fragmentary History of Priscus: Attila, the Huns and the Roman Empire, AD 430–476 (Christian Roman Empire) (trans. Given, J.) (Evolution Publishing, 2005).

[15] Pálffy, G. The Kingdom of Hungary and the Habsburg Monarchy in the Sixteenth Century (trans. DeKornfeld, T. J. & DeKornfeld, H. D.) (Boulder, Center for Hungarian Studies and Publications, Inc. & Columbia University Press, 2009).

[16] Rácz L (2010). The Price of Survival: Transformations in environmental conditions and subsistence systems in Hungary in the age of Ottoman occupation. Hungarian Studies.

[17] Bartosiewicz. J (1997). The Hungarian Grey cattle: a traditional European breed. Anim. Genet. Resour..

[18] Boyd, L. & Houpt, K. A. Przewalski’s Horse: The History and Biology of an Endangered Species (State University of New York Press, 1994).

[19] Balogh I (1958). Pusztai legeltetési rend Debrecenben a XVIII — XIX. században [Extensive grazing regime in Debrecen in the 18th–19th centuries]. Ethn..

[20] Érkövy, A. A 1863. évi aszályosság a Magyar Alföldön [The Drought of 1863 in the Hungarian Plain] (Országos Magyar Gazdasági Egyesület, 1863).

[21] Paládi-Kovács, A. A magyar állattartó kultúra korszakai [Historical Periods of Animal Husbandry in Hungary] (MTA Néprajzi Kutatóintézet, 1993).

[22] Archivio Segreto Vaticano, A. A. Arm. I-XVIII 605. (eds Theiner, A.) 230–232 (Romae Typis Vaticanis, 1859); Hungarian National Archive Diplomatic Collection, MNL OL, DF 289184 (1250-11-11). Translated by Piroska Nagy and found in: Rosenwein, B. Reading the Middle Ages. Vol. 1 (University of Toronto Press, 2007).

[23] Bretschneider, E. Medieval Researches from Eastern Asiatic Sources Vol. 1 331–332 (Kegan Paul, Trench, Trübner & Co. Ltd., 1910).

[24] Laszlovszky, J. Material Remains of the Mongolian Invasion in Hungary and Development-Led Archaeology. Hungarian Archaeology. Spring, 1–3 (2012).

[25] Vargha, M. Hoards, grave goods, jewellery. Objects in hoards and in burial contexts during the Mongol invasion of Central-Eastern Europe (Archaeopress, 2015).

[26] Kralovánszky, A. The Settlement History of Veszprém and Székesfehérvár in the Middle Ages. In: Geravich, L. (ed.) Towns in Medieval Hungary, 51–95 (Akadémiai Kiadó, 1990).

[27] Augsburg University Library, 02/IV.13.2.26-13

[28] Luterbacher J, Pfister C (2015). The year without a summer. Nature Geosci..

[29] Pinke, Z. & Lövei, G. L. Increasing temperature cuts back crop yields in Hungary over the last 90 years. *Glob. Chang. Biol.* doi:10.1111/gcb.13808 (in print) (2017). 10.1111/gcb.13808 28699259

