# Supplementary material for: Climate of doubt: A re-evaluation of Büntgen and Di Cosmo’s environmental hypothesis for the Mongol withdrawal from Hungary, 1242 CE
Source: Sci Rep. 2017 Oct 5;7:12695. doi: 10.1038/s41598-017-12128-6 (PMC5629221; doi:10.1038/s41598-017-12128-6)
Supplement: Supplementary file 1 — Supplementary [file 41598_2017_12128_MOESM1_ESM.pdf]

Supplementary Information

Title of the article: Climate of doubt: a re-evaluation of Büntgen and Di Cosmo's environmental hypothesis for the Mongol withdrawal from Hungary, 1242 CE

Authors: Zsolt Pinke, László Ferenczi, Beatrix F. Romhányi, József Laszlovszky, Stephen Pow

Appendix of Figure 1. Test results of linear models[29,30] between the first-differencesof March–August precipitation sums[31] and the annual yield means of four crops[32] in the period 1921–2010 using Rcmdr package of Rx364 3.2.4 Revised version[33] (n=90; α=0.05). Normality was tested by Shapiro-Wilk normality test and visual inspection of histograms.

	R^2	p-value	R^2	p-value	R^2	p-value	R^2	p-value
	Barley		Oat		Rye		Wheat	
Precipitation (equation)	0.07 (y = 0.0013x + 0.0235)	0.01	0.05 (y = 0.001x + 0.0136)	0.04	0.06 (y = 0.0008x + 0.0096)	0.02	0.05 (y = 0.0013x + 0.0246)	0.04

References

29. Nicholls, N. Increased Australian wheat yield due to recent climate trends. *Nature* **387**, 484–485 (1997).

30. Lobell, D. & Field, C. Global scale climate–crop yield relationships and the impacts of recent warming. *Environ. Res. Lett.* **2**, 014002 (2007).

31. Hungarian Central Statistical Office. Crop yields and harvested area 1921−2010. https://www.ksh.hu/docs/hun/agrar/html/tabl1_4_3_1.html;

(accessed 15. 03. 12).

32. Hungarian Meteorological Service. Monthly precipitation and temperature data 1921−2010. omsz.hu; http://www.met.hu/eghajlat/magyarorszag_eghajlata/eghajlati_adatsorok/; (accessed 15. 07. 16).

33. Fox, J. The R Commander: A Basic-Statistics Graphical User Interface to R. *J. Stat. Softw.* **14**, 1–42 (2005).
